# Glutathione Peroxidase-1 Suppresses the Unfolded Protein Response upon Cigarette Smoke Exposure

**DOI:** 10.1155/2016/9461289

**Published:** 2016-12-13

**Authors:** Patrick Geraghty, Nathalie Baumlin, Matthias A. Salathe, Robert F. Foronjy, Jeanine M. D'Armiento

**Affiliations:** ^1^Division of Pulmonary & Critical Care Medicine, Department of Medicine, State University of New York Downstate Medical Center, Brooklyn, NY, USA; ^2^Department of Cell Biology, State University of New York Downstate Medical Center, Brooklyn, NY, USA; ^3^Division of Pulmonary, Allergy, Critical Care, and Sleep Medicine, University of Miami, Miami, FL, USA; ^4^Center for Pulmonary Disease, Department of Anesthesiology, College of Physicians and Surgeons, Columbia University, New York, NY, USA

## Abstract

Oxidative stress provokes endoplasmic reticulum (ER) stress-induced unfolded protein response (UPR) in the lungs of chronic obstructive pulmonary (COPD) subjects. The antioxidant, glutathione peroxidase-1 (GPx-1), counters oxidative stress induced by cigarette smoke exposure. Here, we investigate whether GPx-1 expression deters the UPR following exposure to cigarette smoke. Expression of ER stress markers was investigated in fully differentiated normal human bronchial epithelial (NHBE) cells isolated from nonsmoking, smoking, and COPD donors and redifferentiated at the air liquid interface. NHBE cells from COPD donors expressed heightened ATF4, XBP1, GRP78, GRP94, EDEM1, and CHOP compared to cells from nonsmoking donors. These changes coincided with reduced GPx-1 expression. Reintroduction of GPx-1 into NHBE cells isolated from COPD donors reduced the UPR. To determine whether the loss of GPx-1 expression has a direct impact on these ER stress markers during smoke exposure,* Gpx*-*1* ^−/−^ mice were exposed to cigarette smoke for 1 year. Loss of* Gpx*-*1* expression enhanced cigarette smoke-induced ER stress and apoptosis. Equally, induction of ER stress with tunicamycin enhanced antioxidant expression in mouse precision-cut lung slices. Smoke inhalation also exacerbated the UPR response during respiratory syncytial virus infection. Therefore, ER stress may be an antioxidant-related pathophysiological event in COPD.

## 1. Introduction

Chronic obstructive pulmonary disease (COPD) is the third leading cause of death in the US [1], with cigarette smoking being the most important environmental risk factor. Cigarette smoke inhalation alters the expression profile of oxidants and antioxidants in the lungs and produces an enormous oxidant burden [2]. Antioxidant enzymes counter this oxidative stress [2] and deter lung inflammation responses by targeting multiple signaling pathways [3]. Detoxifying reactive oxygen species (ROS) is a therapeutic strategy to limit tissue damage in cigarette smoke-induced diseases [3]. Recently, cigarette smoke-mediated oxidative stress was shown to induce endoplasmic reticulum (ER) stress [4]. However, the ability of antioxidants to counter ER stress has not been fully characterized.

It is well established that cigarette smoke induces ER stress which activates the unfolded protein response (UPR) [5–9]. However, COPD is a complex heterogeneous disease and the significance and intensity of the UPR during the disease is unknown. The UPR is a complex stress response program that modulates multiple cellular responses and survival, via regulation of protein synthesis, folding, and degradation [10]. Three major pathways of the UPR have been characterized: (i) PKR-like ER kinase (PERK)/eIF2*α*/activating transcription factor (ATF) 4/CHOP, (ii) inositol-requiring enzyme 1 (IRE1)/X-box binding protein 1 (XBP1), and the (iii) ATF6 pathway [11]. These pathways regulate ER chaperone responses and reduce protein translation following cellular stress [12]. However, persistent ER stress results in significant expression of the proapoptotic gene C/EBP homologous protein (CHOP), resulting in cell death [11]. We previously demonstrated that smoke exposure triggers a minor ER stress response in primary cells and rodent animal models [5]. However, a secondary insult may be required to provoke a significant smoke-induced UPR.

Our group demonstrated that overexpression of glutathione peroxidase- (GPx-) 1, a member of the selenoprotein family, prevents cigarette smoke-induced air space enlargement in mice [13]. GPx-1 is the most abundant GPx isoform in eukaryotic cells and deficiency of this enzyme can lead to endothelial dysfunction [14] and apoptosis [15]. GPx-1 deficiency has also been implemented as a contributor to atherosclerosis [16].* Gpx*-*1* deficient mice exposed to cigarette smoke are more susceptible to cigarette smoke-induced lung inflammation and emphysema [13, 17]. Oxidative stress induces ER stress [4] and increased expression of ER stress markers is observed in the lungs of smokers [8]. GPx-1 expression, however, is reduced in COPD lungs [13]. Thus, we speculate that GPx-1 could modulate ER stress responses linked to the pathogenesis of COPD.

In view of the potential association between GPx-1 and cigarette smoke-mediated UPR, we explored whether the loss of GPx-1 expression enhanced the UPR that contributes to lung cell injury and death. Using normal human bronchial epithelial (NHBE) cells from nonsmokers, smokers, and COPD subjects, we found that ER stress markers were significantly elevated in cells isolated from COPD subjects and this increase coincided with reduced GPx-1 expression. Reintroducing GPx-1 into these cells blunted the UPR. To determine if GPx-1 depletion in the lung directly enhances ER stress,* Gpx-1* ^−/−^ mice were exposed to cigarette smoke for 1 year. Interestingly, the loss of GPx-1 expression activated all three branches of the UPR, PERK/eIF2*α*/ATF4/CHOP, IRE1/XBP1, and ATF6. This UPR coincided with elevated lung cell death in* Gpx-1* ^−/−^ mice following smoke exposure. Interestingly, precision-cut lung slices (PCLS) from mice had elevated GPx proteins following induction of ER stress. These findings indicate that the altered GPx-1 expression in COPD lungs contributes to heightened ER stress. In addition, early induction of ER stress induces an antioxidant response to counter oxidative stress, thereby limiting the UPR.

## 2. Materials and Methods

### 2.1. Human Primary Airway Cells

NHBE cells from nonsmokers, smokers, and COPD patients were isolated from human lungs. Lungs were obtained from organ donors whose lungs were rejected for transplant (see Table 1 for demographics). Consent for research was obtained by the Life Alliance Organ Recovery Agency of the University of Miami. All consents were IRB-approved and conformed to the Declaration of Helsinki. For lungs from donors with COPD, the diagnosis was listed in the chart before the death of the donor and we confirmed the macropathological presence of emphysema in these lungs. All COPD subjects had a significant smoking history. NHBE cells isolated from nonsmokers, smokers, and COPD subjects were dedifferentiated through expansion and redifferentiated at an air liquid interface (ALI) on 24 mm T-clear filters (Costar Corning, Corning, NY, USA) at 37°C, 5% CO_2_, as previously described [18]. Cells were collected for protein and RNA analysis. CD45 and CD11C expressions were analyzed which determined a low level of inflammatory-cell contamination and confirmed NHBE cell purity. Fully differentiated NHBE cells from nonsmokers were also exposed to cigarette smoke using a Vitrocell VC-10 smoking robot (Vitrocell Systems GMBH, Waldkirch, Germany). Four cigarettes were smoked according to ISO standard 3308: six puffs per cigarette with a 35 mL volume per puff and a waiting time between each puff of 60 seconds. NHBE cells were exposed every second day, on three separate days, to 4 cigarettes. RNA was extracted from the NHBE cells for quantitative PCR (qPCR) analysis. A subset of NHBE cells from COPD subjects was protein transfected 2 *μ*g human GPx-1 protein or human albumin (both from Signal Aldrich) using Pierce transfection reagent (ThermoFisher Scientific) as previously described [13, 19]. RNA and protein were collect 24 hours later. Human RSV strain A2 (ATCC, Manassas, VA; #VR-1540) was infected in NHBE cells as previously described [20]. RNA and protein were collect 24 hours later.

### 2.2. Animal Models

*Gpx-1* ^−/−^ mice were bred in C57BL/6 × CBA/J background. Eight-week-old wild type and* Gpx-1* ^−/−^ mice were used for all experiments. All mice were maintained in a specific pathogen-free facility at Columbia University. Both male and female mice, 8-week-old, were used at the initiation point for all experiments and each experimental parameter had at least 10 animals per group. Mice were exposed to cigarette smoke in a chamber (Teague Enterprises, Davis, CA, USA) for four hours a day, five days per week at a total particulate matter concentration of 80 mg/m^3^. Smoke exposure was continued for 1 year. The University of Kentucky reference research cigarettes 3R4F (Lexington, KY, USA) were used to generate cigarette smoke. Another group of wild type animals was infected with 1 × 10^6^ pfu of RSV following 6-month exposure to room air or cigarette smoke. The institute approved all experiments for Animal Care and Use Committee of Columbia University. This study was performed in strict accordance with the recommendations in the Guide for the Care and Use of Laboratory Animals of the National Institutes of Health and Institutional Animal Care and Use Committee (IACUC) guidelines.

### 2.3. Precision-Cut Lung Slices (PCLS)

Mouse precision-cut lung slices (PCLS) were prepared as previously described [21, 22]. Briefly, mice were euthanized, the trachea was cannulated, and the animal was exsanguinated by cutting the jugular vein. The lungs were filled through the cannula with 1.5 mL low melting-point agarose solution (1.5% final concentration of agarose in PBS). Lungs were placed on ice for 15 minutes to solidify the agarose. Lobes were separated and tissue cores were prepared of the individual lobes, after which the lobes were sliced at a thickness of 300 *μ*m using a Krumdieck tissue slicer (Alabama Research and Development, Munford, AL, USA) in Earle's balanced salt solution (Sigma Aldrich). Tissue slices were incubated in Dulbecco's modified eagle's medium/nutrient mixture F-12 HAM solution (Sigma Aldrich) at 37°C in a humid atmosphere under 5% CO_2_/95% air. To remove agarose and cell debris, slices were washed every 30 minutes for 2 hours. PCLS were incubated in DMEM supplemented with penicillin (100 U/mL) and streptomycin (100 *μ*g/mL) (Gibco® by Life Technologies). Slices were cultured at 37°C in a humidified atmosphere under 5% CO_2_/95% air in 6-well tissue culture plates, using 3 slices per well. Slices were treated with 1 *μ*M tunicamycin (Sigma Aldrich) for 24 hours. To assess the viability of the PCLS subjected to tunicamycin, lactate dehydrogenase (LDH) released from the PCLS into the incubation medium was analyzed. Maximal LDH release was determined by lysing 3 slices with 1% Triton X-100 for 30 minutes at 37°C. LDH release was determined using an assay form Sigma Aldrich.

### 2.4. qPCR Analysis

Total RNA was isolated from cells or mouse lung tissue using the Qiagen RNeasy Mini Kit as described by manufactures. Gene transcript levels of mouse and human specific CHOP, ATF4, XBP1, GRP78, GRP94, EDEM1, GPx-1, GPx-2, GPx-3, GPx-4, IL-6, and ACTB were quantified by real-time PCR with the use of an Bio-Rad CFX384 real-time system (Bio-Rad). TaqMan® Gene Expression Assays were purchased from Applied Biosystems (see Table 2 for details). Data is represented as relative quantification (RQ) corrected to ACTB. XBP1 splicing was also examined in NHBE cells using the following primers: 5′-TTA CGA GAG AAA ACT CAT GGC-3′ and 5′-GGG TCC AAG TTG TCC AGA ATG C-3′. XBP1 PCR products were resolved and run on a 2.5% agarose gel [23]. 289 and 286 base pair amplicons were generated from unspliced and spliced XBP1, respectively. Percent of XBP1 slicing was examined by densitometry analysis of the unspliced (XBP1u) and spliced (XBP1s) amplicons of XBP1, using Bio-Rad Laboratories Image Lab software (version 4.0, build 16).

### 2.5. Immunoblotting

Cell monolayers were removed by scrapping in cold phosphate-buffered saline and resuspended in 100 *μ*L of protein lysis buffer (50 mM HEPES, pH 7.5, 150 mM NaCl, 1% Triton X-100, 1% glycerol, 1 mM EDTA, 10 mM NaF, 2 *μ*g/mL leupeptin, 1 *μ*g/mL pepstatin A, 10 mM Na_3_VO_4_, and 1 mM phenylmethylsulfonyl fluoride), and 20 *μ*g of protein was separated on 12% SDS-polyacrylamide gels and transferred to nitrocellulose membranes. Rabbit antibodies against CHOP (Cell Signaling; #5554), ER degradation-enhancing *α*-mannosidase-like (EDEM) (Santa Cruz Biotechnology; sc-27389), pho-eIF2*α* (Ser51) (Cell Signaling; #9721), eIF2*α* (Cell Signaling; #9722), pho-PERK (Thr980) (Cell Signaling; #3179), PERK (Cell Signaling; #5683), XBP-1 (Cell Signaling; #12782), IRE1*α* (Cell Signaling; #3294), BiP/GRP78 (Cell Signaling; #3183), GRP94 (Cell Signaling; #2104), ATF4 (Cell Signaling; #11815), ATF6 (Abcam; #ab11909), GPx-1 (Cell Signaling; #3206), GPx-2 (Abcam; #ab140130), GPx-3 (Abcam; #ab27325), GPx-4 (Cell Signaling; #2104), and *β*-actin (Cell Signaling; #4970) were detected with enhanced chemiluminescence reagents (Pierce). Chemiluminescence detection was performed using the Bio-Rad Laboratories Molecular Imager ChemiDoc XRS+ imaging system. Densitometry was performed on each target and represented as a ratio of pixel intensity compared to total protein or *β*-actin, using Bio-Rad Laboratories Image Lab software (version 4.0, build 16).

### 2.6. Statistical Analysis

Data are expressed as dot plots with the means ± SEM highlighted. Differences between two groups were compared by Student's *t* test (two-tailed). Experiments with more than 2 groups were analyzed by 2-way ANOVA with Tukey's* post hoc* test analysis. *p* values for significance were set at 0.05 and all significant changes were noted with *∗*. All analysis was performed using GraphPad Prism Software (Version 6.0h for Mac OS X).

## 3. Results

### 3.1. NHBE Cells Isolated from COPD Donors Express More ER Stress Markers than Cells from Smokers

**Figure 1:**
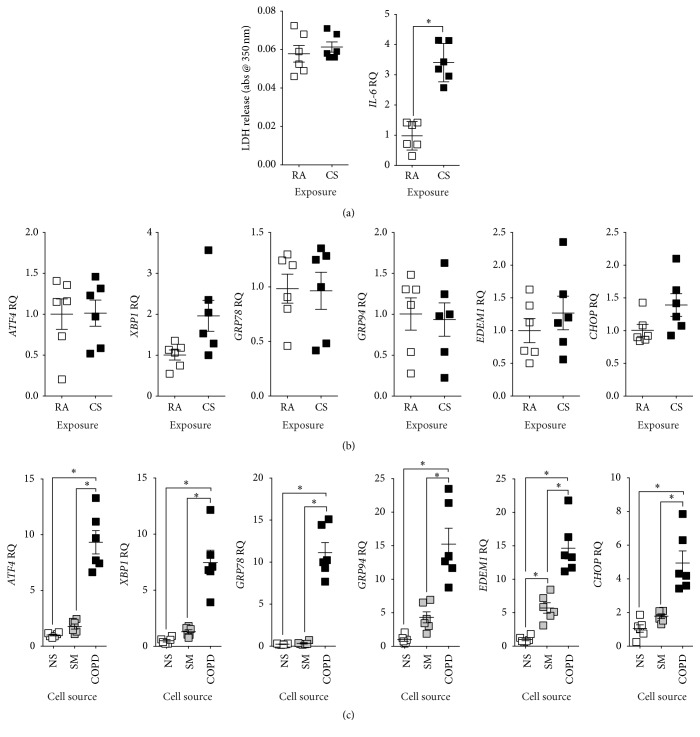
NHBE cells isolated from COPD donors have enhanced ER stress responses compared to nonsmokers and smokers. (a) Fully differentiated NHBE cells from nonsmoking individuals (*n* = 6) exposed to room air (RA) or cigarette smoke (CS) from 4 cigarettes every second day (3 exposures) using a Vitrocell VC-10 smoking robot. LDH release into media and* IL-6* gene expression were examined. (b) Gene expression of* ATF4*,* XBP1*,* GRP78*,* GRP94*,* EDEM1,* and* CHOP* was examined. (c) Fully differentiated NHBE cells from nonsmoker (NS), smoker (SM), and COPD (COPD) individuals (*n* = 6 donors per group) were examined for gene expression of* ATF4*,* XBP1*,* GRP78*,* GRP94*,* EDEM1,* and* CHOP*. Dot plots are represented as relative quantification (RQ) compared to ACTB expression and shown as the mean ± SEM, where each measurement was performed on 3 independent days on 6 donors/group. *∗* denotes *p* value < 0.05, when comparing both treatments connected by a line, determined by Student's *t*-test (2 groups) or 2-way ANOVA with Tukey's* post hoc* test (>2 groups).

**Figure 2:**
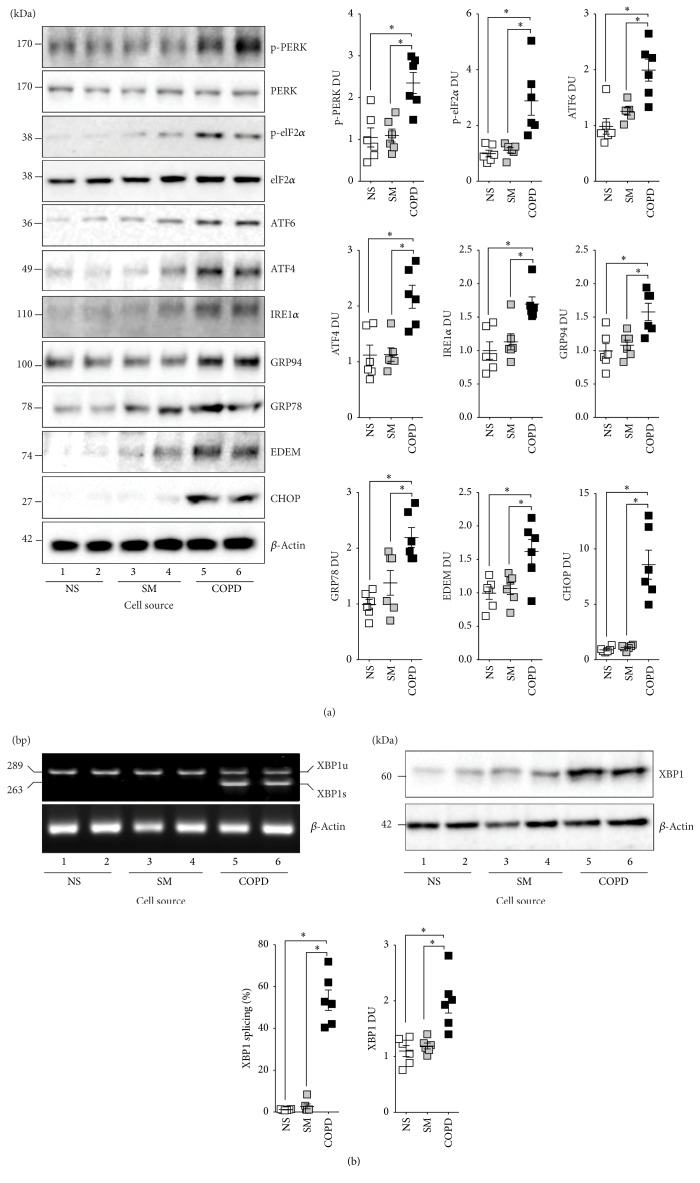
NHBE cells isolated from COPD donors have enhanced ER stress protein responses compared to nonsmokers and smokers. Protein was collected from fully differentiated NHBE cells from nonsmokers (NS), smokers (SM), and COPD (COPD) individuals (*n* = 6 donors per group). (a) Protein expression of ATF6, ATF4, GRP94, GRP78, EDEM, CHOP, and *β*-actin was examined by immunoblots. Phosphorylation of elF2 and PERK was also determined. (b) XBP1 splicing was examined on XBP1 amplified cDNA from NHBE cells from nonsmoker (NS), smokers (S), and COPD (COPD) individuals. Protein expression of XBP1 and *β*-actin was examined by immunoblots. (a)-(b) For each blot or gel, every lane represents an individual cell donor. Densitometry analysis was performed of XBP1s from DNA gels and other targets by analyzing immunoblots. XBP1 slicing was scored as percent of XBP1s of total XBP1. Dot plots are represented as densitometry units (DU) of pixel intensity expressed as a ratio to *β*-actin or total elF2 and PERK. Data are shown as mean ± SEM, where each measurement was performed on 3 independent days on 6 donors/group. *∗* denotes *p* value < 0.05, when comparing both treatments connected by a line, determined by 2-way ANOVA with Tukey's* post hoc* test (>2 groups).

Our group previously demonstrated that undifferentiated NHBE cells have an increased trend in ER stress upon exposure to cigarette smoke extract (CSE) [5]. To examine whether fully differentiated NHBE cells cultured at the air liquid interface (ALI) have an UPR to smoke, NHBE cells from nonsmokers were exposed to 0 (room air, RA) or repeat exposure to four cigarettes (CS) using a Vitrocell VC-10 smoking robot (Figures 1(a)-1(b)). Repeat exposures were performed to maximize smoke stimuli without inducing apoptosis, determined by LDH release assays (Figure 1(a)). Gene expression of IL-6 was utilized as a positive control for sufficient exposure to smoke [20] (Figure 1(a)). CD45 and CD11C expressions were analyzed but detection was below significant amplification levels thereby confirming low levels of inflammatory-cell contamination (data not shown). Gene expression levels of* ATF4, XBP1, GRP78, GRP94, EDEM1,* and* CHOP* were examined. These targets are readouts for the three major pathways of the UPR. No ER stress marker was significantly altered following smoke exposure (Figure 1(b)), as we previously described in submerged cultured NHBE cells [5]. However, when comparing the same ER stress markers in NHBE cells isolated from nonsmokers, smokers, and COPD donors, expressions of* ATF4*,* XBP1*,* GRP78*,* GRP94*,* EDEM1,* and* CHOP* were all increased in cells isolated from COPD subjects (Figure 1(c)).* EDEM1* gene expression was significantly enhanced in cells isolated from smokers (Figure 1(c)). There were increased trend changes for ER stress markers in cells from smokers. Protein analysis also confirmed increased expression of ATF4, IRE1*α*, GRP78, GRP94, EDEM, and CHOP in cells isolated from COPD subjects (Figure 2(a)). Equally, elevated phosphorylation of eIF2 and PERK was observed only in cells isolated from COPD donors (Figure 2(a)).

XBP1 coordinates the adaptive UPR by playing a vital role in maintaining the ER function. Gene expression results showed that NHBE cells isolated from COPD subjects had enhanced XBP1 mRNA splicing compared to cells from nonsmokers and smokers (Figure 2(b)), demonstrating active XBP1 signaling; Protein analysis also confirmed increased expression of XBP1 in cells isolated from COPD donors (Figure 2(b)). Overall, the disease-state predisposes NHBE cells to enhanced ER stress. On the other hand, acute smoke exposure has only a minor impact on ER stress.

### 3.2. GPx-1 Regulates CHOP Expression in NHBE Cells Isolated from COPD Donors

**Figure 3:**
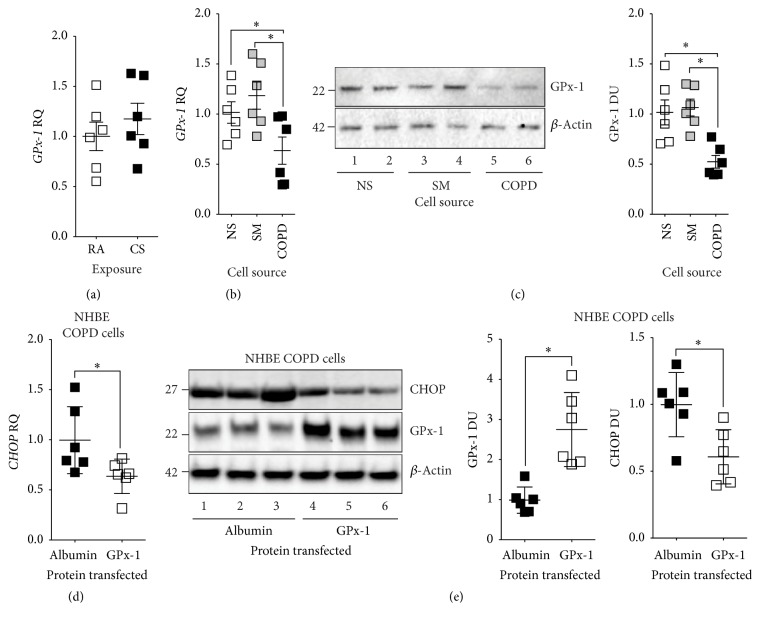
Reintroducing GPx-1 into NHBE cells isolated from COPD donors subdues the UPR. (a) Gene expression of* GPx-1* was determined in fully differentiated NHBE cells from nonsmoking individuals (*n* = 6) exposed to room air (RA) and cigarette smoke (CS; from 4 cigarettes every second day (3 exposures)) using a Vitrocell VC-10 smoking robot (b) RNA and (c) protein was analyzed for GPx-1 expression from fully differentiated NHBE cells from nonsmoker (NS), smokers, (SM) and COPD (COPD) individuals (*n* = 6 donors per group). (d) NHBE cells isolated from COPD subjects were transfected with albumin or GPx-1 protein and* CHOP* expression was determined by qPCR. (e) Immunoblots and corresponding densitometry analysis for CHOP, Gpx-1, and *β*-actin from NHBE cells from COPD subjects following albumin or GPx-1 protein transfection. In each immunoblot, every lane represents an individual cell donor. Data are shown as mean ± SEM, where each measurement was performed on 3 independent days on 6 donors/group. *∗* denotes a *p* value < 0.05, when comparing both treatments connected by a line, determined by Student's *t*-test (2 groups) or 2-way ANOVA with Tukey's* post hoc* test (>2 groups).

Since oxidative stress induces ER stress [4], GPx-1 expression is reduced in the COPD lungs [13], and GPx-1 deficiency increases susceptibility to cigarette smoke-induced emphysema [13, 17], we examined whether GPx-1 expression was altered in fully differentiated NHBE cells isolated from nonsmokers, smokers, and COPD donors (Figure 3). GPx-1 expression was unchanged in NHBE cells from nonsmokers when exposed to cigarette smoke (Figure 3(a)). NHBE cells from nonsmokers and smokers expressed comparable levels of GPx-1 (Figures 3(b)-3(c)). However, cells isolated from COPD subjects had significantly reduced GPx-1 expression, confirmed by q-PCR (Figure 3(b)) and immunoblots (Figure 3(c)). Therefore, the disease-state predisposes NHBE cells to subdued GPx-1 expression.

To determine whether restoring GPx-1 levels in NHBE cells from COPD subjects would reverse heightened UPR, we protein-transfected GPx-1 protein into NHBE cells from COPD subjects. Albumin was transfected as a negative control. Transfection of GPx-1 significantly reduced CHOP gene (Figure 3(d)) and protein (Figure 3(e)) expression in NHBE cells from COPD subjects. Therefore, GPx-1 is a potent regulator of the UPR in the lungs.

### 3.3. Viral Exacerbations of the Lung Enhance the UPR

**Figure 4:**
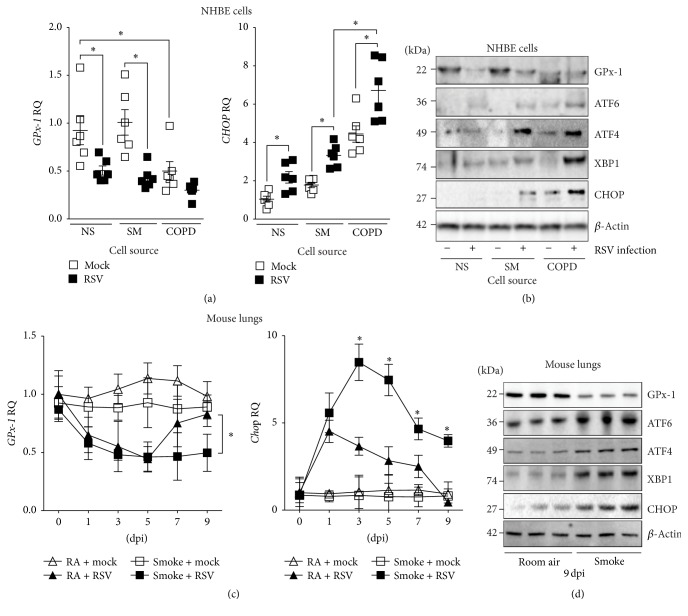
RSV infection enhances the UPR in the lung. (a) GPx-1 and CHOP gene expression were determined in NHBE cells isolated from nonsmoker (NS), smokers (SM), and COPD (COPD) individuals (*n* = 6 donors per group) infected with mock or RSV and analyzed by qPCR. (b) Protein expression of GPx-1, ATF6, ATF4, CHOP, and *β*-actin was examined by immunoblots. (c) Wild type mice were exposed to cigarette smoke or room air for six months and subsequently infected with 1 × 10^6^ pfu of RSV. Animals were euthanized at 0, 1, 3, 5, 7, and 9 days after infection (dpi) and* Gpx-1* and* Chop* expression were determined by qPCR. (d) Protein expression of GPx-1, ATF6, ATF4, CHOP, and *β*-actin was examined by immunoblots. Data are shown as mean ± SEM, where each measurement was performed on 3 independent days. *∗* denotes a *p* value < 0.05, when comparing both treatments connected by a line or the same infection day, determined by Student's *t*-test (2 groups) or 2-way ANOVA with Tukey's* post hoc* test (>2 groups).

To determine whether a second environmental exposure could alter the UPR in our models, we infected NHBE cells and mice with respiratory syncytial virus (RSV). Viral infections have been implicated in the pathogenesis of COPD exacerbations [24, 25] and also trigger the UPR [26]. RSV infected reduced GPx-1 expression and significantly enhanced CHOP expression in NHBE cells from all subject groups (Figures 4(a)-4(b)). Similar changes to CHOP and GPx-1 expression were observed in the lungs of mice infected with RSV (Figures 4(c)-4(d)). Importantly, prior exposure to cigarette smoke enhanced the UPR in animals also infected with RSV compared to infected animals exposed to room air (RA) (Figure 4(d)). Therefore, the lungs of smokers and COPD subjects are likely to be more sensitive to viral infection induced ER stress, which may impact disease progression.

### 3.4. *Gpx-1* ^−/−^ Mice Have Heightened ER Stress and Apoptosis following Cigarette Smoke Exposure

**Figure 5:**
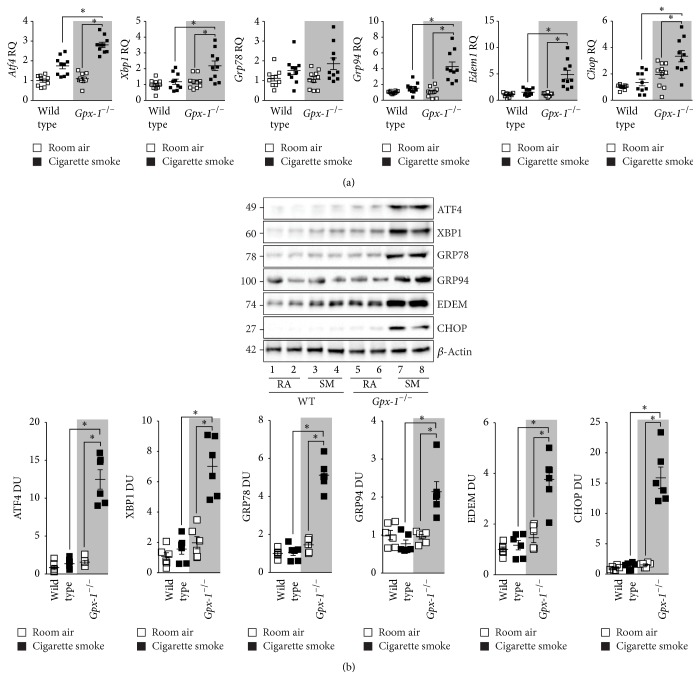
*Gpx-1* deficient mice have heightened ER stress in their lungs following exposure to cigarette smoke.* Gpx-1* ^−/−^ and wild type mice were exposed to cigarette smoke daily for 1 year. (a) Lung gene expression of* Chop*,* Atf4*,* Edem1*,* Grp78*,* Grp94,* and* Xbp1* was examined. (b) Immunoblots were performed of whole lung protein for CHOP, ATF4, EDEM, GRP78, GRP94, and XBP1. Dot plots are represented as (a) relative quantification (RQ) compared to ACTB expression or (b) densitometry units (DU) of pixel intensity expressed as a ratio to *β*-actin. Every lane represents an individual mouse. Data are shown as mean ± SEM, where each measurement was performed on 3 independent days on 6 donors/group. *∗* denotes a *p* value < 0.05, when comparing both treatments connected by a line, determined by 2-way ANOVA with Tukey's* post hoc* test (>2 groups).

To determine whether the loss of GPx-1 expression directly influences ER stress following inhalation of cigarette smoke* in vivo*, we examined ER stress markers in* Gpx-1* ^−/−^ mice and their wild type littermates exposed to cigarette smoke for 1 year. We previously demonstrated that loss of* Gpx-1* expression in mice results in enhanced air space enlargement and inflammation following long-term cigarette smoke exposure [13]. Expression levels of* Atf4*,* Xbp1*,* Grp78*,* Grp94*,* Edem1,* and* Chop* were examined in* Gpx-1* ^−/−^ and wild type mice. Long-term exposure to cigarette smoke did not significantly enhance ER stress marker expression in the lungs of wild type mice (Figure 5(a)). We previously observed similar findings in wild type mice [5]. However,* Gpx-1* ^−/−^ mice exposed to cigarette smoke had enhanced gene expression of* Atf4*,* Xbp1*,* Grp78*,* Grp94*,* Edem1,* and* Chop* (Figure 5(a)). Equally, loss of* Gpx-1* expression resulted in elevated lung tissue protein levels of ATF4, XBP1, GRP78, GRP94, EDEM, and CHOP following smoke exposure (Figure 5(b)). Densitometry analysis confirmed significant increases in lung levels of ATF4, XBP1, GRP78, GRP94, EDEM, and CHOP in* Gpx-1* ^−/−^ mice exposed to cigarette smoke (Figure 5(b)).

**Figure 6:**
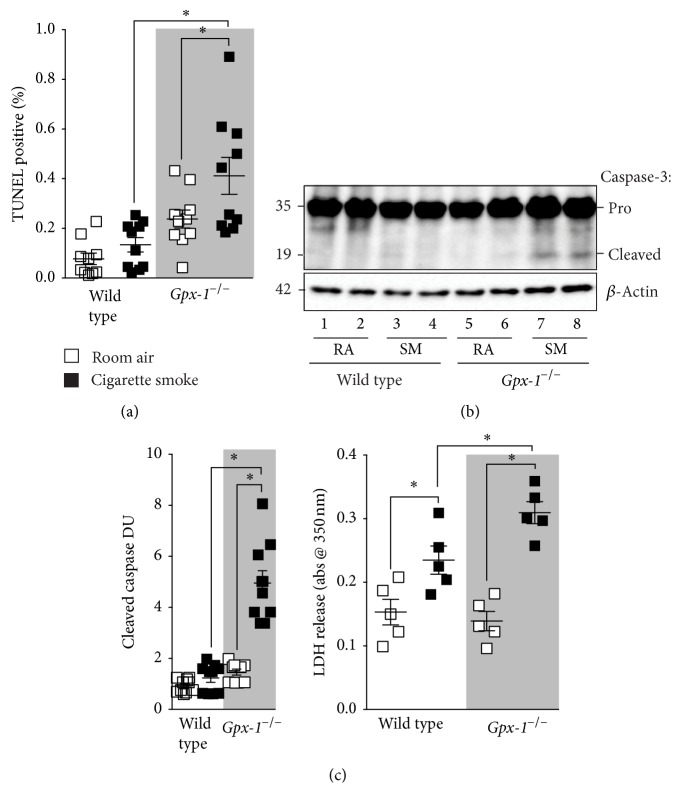
*Gpx-1* deficient mice have heightened ER stress in their lungs following exposure to cigarette smoke.* Gpx-1* ^−/−^ and wild type mice were exposed to cigarette smoke daily for 1 year. (a) TUNEL analysis was performed on lung tissue from each mouse group. (b) Enhanced lung tissue caspase-3 cleavage coincided with (c) elevated LDH into BALF of* Gpx-1* ^−/−^ exposed to cigarette smoke. Every lane in (b) represents an individual mouse and densitometry units (DU) of pixel intensity expressed as a ratio to total caspase-3 levels. Dot plots are represented as mean ± SEM, where each measurement was performed on 3 independent days on 6 donors/group. *∗* denotes a *p* value < 0.05, when comparing both treatments connected by a line, determined by 2-way ANOVA with Tukey's* post hoc* test (>2 groups).

Prolonged activation of CHOP by ER stress can result in cellular apoptosis. Increased structural and immune cell apoptosis is also observed in COPD lungs [27]. Therefore, we examined whether* Gpx-1* ^−/−^ mice exposed to cigarette smoke had elevated apoptosis.* Gpx-1* ^−/−^ mice exposed to cigarette smoke had enhanced lung cell apoptosis, observed by TUNEL, caspase-3 cleavage, and lactate dehydrogenase (LDH) release assays (Figure 6).* Gpx-1* ^−/−^ mice exposed to cigarette smoke exhibited the highest frequency of TUNEL positive cells (Figure 6(a)). Enhanced caspase-3 cleavage was observed in* Gpx-1* ^−/−^ mice exposed to cigarette smoke (Figure 6(b)). Additionally, elevated levels of LDH were observed in the BALF of* Gpx-1* ^−/−^ mice exposed to cigarette smoke compared to the other groups (Figure 6(c)), which indicates enhanced cell membrane damage in the lung. Therefore, enhanced apoptosis in the lungs could contribute to lung remodelling and failure to clear apoptotic cells could contribute to lung inflammation.

### 3.5. Triggering the UPR Enhances GPx-1, GPx-2, and GPx-4 Expression in Mouse Precision-Cut Lung Slices

**Figure 7:**
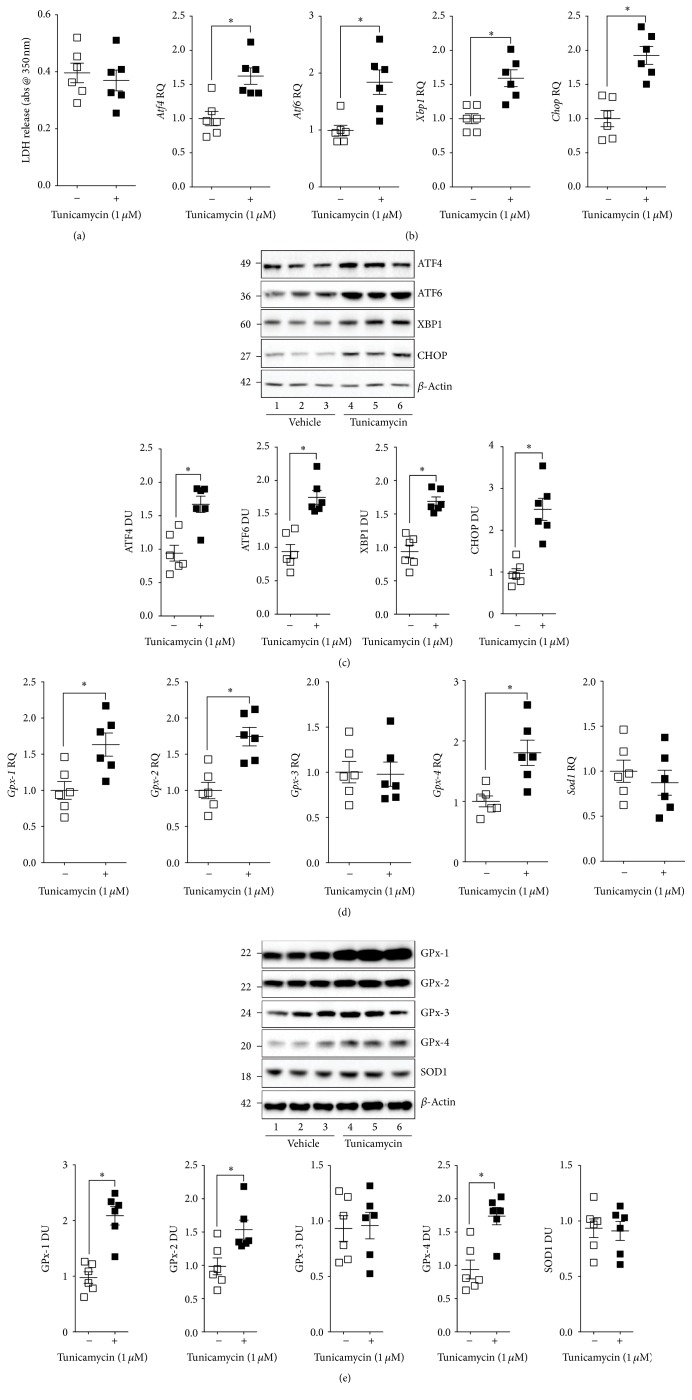
Effect of ER stress-induced tunicamycin on the antioxidant expression profile in mouse precision-cut lung slices (PCLS). PCLS were obtained from wild type mice and were exposed to tunicamycin (1 *μ*M) for 24 hours. (a) LDH release into media and (b)* Chop*,* Atf4*,* Atf6,* and* Xbp1* gene expression were examined. (c) Immunoblots were conducted for* Chop*,* Atf4*,* Atf6, Xbp1, andβ*-actin. (d)* GPx-1, GPx*-*2, GPx*-*3, GPx*-*4,* and* Sod1* were quantified by qPCR and (e) immunoblots analysis. Every lane represents an individual mouse. Dot plots are represented as relative quantification (RQ) compared to ACTB expression or densitometry units (DU) of pixel intensity expressed as a ratio to *β*-actin. Data are shown as the mean ± SEM, where each measurement was performed on 3 independent days on 6 donors/group. *∗* denotes a *p* value < 0.05, when comparing both treatments connected by a line, determined by Student's *t*-test (2 groups).

In* Gpx-1* and* Gpx-2* double knockout mice, apoptotic cells are increased in ileal crypts [28], suggesting that GPx proteins regulate apoptosis. However, the effect of ER stress on GPx proteins has not been directly investigated. To determine the effect of ER stress on GPx-1 expression, mouse precision-cut lung slices (PCLS) were exposed to the ER stress inducer, tunicamycin, for 24 hours. The concentration of tunicamycin tested did not induce LDH release from the PCLS (Figure 7(a)), indicating no induction of apoptosis. Tunicamycin induced all three branches of the UPR and CHOP expression was observed in PCLS (Figures 7(b)-7(c)). Tunicamycin enhanced gene (Figure 7(b)) and protein (Figure 7(c)) expression of ATF4, XBP1, ATF6, and CHOP in PCLS. Interestingly, tunicamycin induced GPx-1, GPx-2, and GPx-4 gene (Figure 7(d)) and protein (Figure 7(e)) expression in PCLS. Therefore, an acute ER stress induces the expression of antioxidants to counter further oxidant and ER stress.

## 4. Discussion

**Figure 8:**
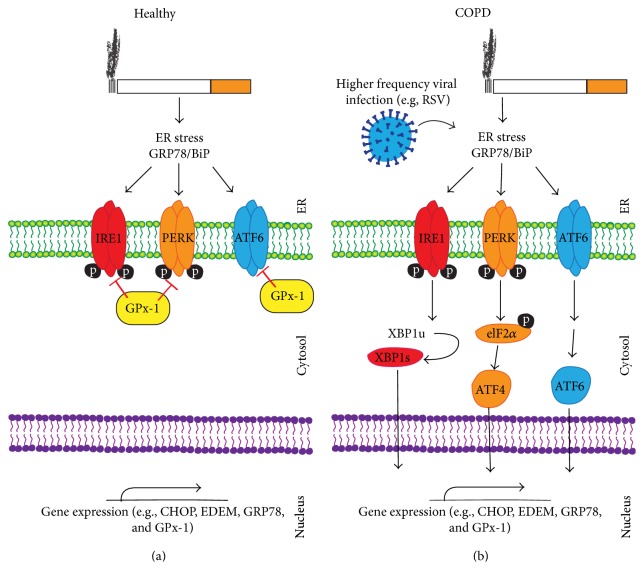
Possible signaling mechanism for GPx-1 regulation of the UPR. Evidence presented in this study indicates that following smoke exposure GPx-1 prevents ER stress (a). However, in the disease-state GPx-1 expression is subdued and results in enhanced UPR (b). RSV infection significantly contributes to reduced GPx-1 expression that coincides with enhanced UPR.

Cigarette smoking is the most relevant environmental risk factor associated with the development of COPD. However, smoke inhalation studies are problematic as long-term smoke exposure is required to trigger disease formation in animal models and a secondary event may be required to mimic the human disease-state. Here we observed that the loss of GPx-1 expression enhances cigarette smoke-induced ER stress. GPx-1 regulates the UPR following smoke exposure and we found that the expression of GPx-1 itself was triggered by an acute ER stress stimulus. NHBE cells isolated from COPD donors expressed significantly less GPx-1, which coincides with elevated UPR. Reintroducing GPx-1 protein into NHBE cells isolated from COPD donors reduced the UPR. RSV infection contributes to loss of lung GPx-1 expression, which is exaggerated in lungs exposed to smoke and coincides with elevated UPR.* Gpx-1* ^−/−^ mice exhibited greater UPR and subsequent enhanced apoptosis following long-term cigarette smoke exposure. Interestingly, triggering an acute ER stress in the lungs of mice induces a potent antioxidant response. This antioxidant response is diminished in the COPD lungs [29], which may explain the heightened UPR observed in NHBE cells isolated from COPD subjects. The exact role of this heightened UPR on the progression of COPD still remains to be fully determined. However, we have established that loss of GPx-1* in vivo* leads to a marked increased in all three branches of the UPR (see Figure 8 for proposed signaling scheme) and this coincides with enhanced apoptosis and lung tissue destruction in mice [13]. Our results suggest that GPx-1 significantly regulates the UPR in COPD and enhancing GPx-1 expression may be feasible means of offsetting the UPR and lung injury responses that drive the onset and progression of this disease.

Multiple studies utilizing the* Gpx-1* ^−/−^ and transgenic mice demonstrated the protective role of GPx-1 in countering oxidative injury and cell death mediated by ROS [13, 30]. GPX-1 activity also affects protein kinase phosphorylation [31] and oxidant-mediated activation of NF-*κ*B [32]. In this current study, GPx-1 was significantly reduced in NHBE cells isolated from COPD subjects compared to nonsmokers and smokers. Others have reported that the alteration of GPx-1 expression does not affect the mRNA or activity expression of other selenoproteins [33], which suggests no compensation expression of other selenoproteins following loss of GPx-1 expression. Currently the mechanism by which cigarette smoke regulates GPx-1 expression is not fully elucidated but considering our GPx-1 reintroduction data further analysis of GPx-1 regulation is critical. GPx-1 expression and activity have been reported to be regulated by Nrf2 [34], the transcription factor TFAP2C [35], CpG methylation of the GPx-1 promoter [35], Bcr-Abl/mTOR [36], selenium [37], estrogen [38], adenosine [39], Sec-insertion sequence (SECIS) factors [40], EGFR [41], and homocysteine [42]. Specifically, within the lung during smoke exposure, Singh et al. show elevated GPx-1 expression in the lungs following one-month cigarette smoke exposure that was regulated by Nrf2 [34]. However, Nrf2 expression is lost in COPD subjects suggesting that this secondary event could result in reduced GPx-1 expression and heightened ER stress. Loss of Nrf2 in mice results in enhanced susceptibility to cigarette smoke [43, 44] and elastase [45] induced emphysema in mice. However genomic studies in* Nrf2* ^−/−^ mouse samples suggest that Nrf2 may regulate other GPx genes but not GPx-1 [46]. Whether RSV infection alters regulation of GPx-1 expression in a similar manner to chronic smoke is unknown. Interestingly, the UPR upon RSV infection can counter viral proliferation [26]. Further studies on the regulation of GPx-1 in smoke exposure and COPD and the significance of ER stress in the lungs are required. This will be a major area for our future work.

Since GPx-1 expression regulates all three branches of the UPR, GPx-1 may affect a common mediator of the UPR or each branch individually. Dissociation of GRP78/BiP upon ER stress is required for all three branches of the UPR. GPx-1 expression directly regulated the gene expression of GRP78. However, whether GPx-1 impacts on GRP78 dissociation during ER stress is unknown. Nrf2 interacts directly with PERK [47] and may play a major role in GPx-1 expression thereby regulating the UPR. The ER stress inducer, thapsigargin, induces Nrf2 protein production in 16-HBE cells [47], which suggests that the UPR induces a Nrf2 response to reverse ER stress. We observe a similar effect on GPx-1 expression in PCLS following tunicamycin treatment. Equally, XBP1 regulates several antioxidants, including catalase, SOD1, and thioredoxin TRX1 [48]. However, XBP1 does not regulate GPx proteins [48] but XBP1 expression is regulated by GPx-1. The XBP1 regulated gene,* EDEM1*, was also enhanced in* Gpx-1* ^−/−^ mice exposed to smoke, which further confirms that GPx-1 regulation of XBP1 signaling. ATF6 requires translocation to the Golgi to undergo cleavage and subsequent translocation to the nucleus to act as a transcription factor [49]. Whether GPx-1 modulates this signaling has yet to be determined. This crosstalk between antioxidant signaling and the UPR is partially lost in COPD and may play a critical step in the pathogenesis of this disease.

The role of the UPR on apoptosis is dependent on the stimulus, exposure duration, and intensity of this signaling. Loss of GPx-1 expression directly impacts cell death and cell death is an important factor in COPD progression [50]. Smoke-induced apoptosis has been associated with several processes, such as ceramide signaling [51], damage-associated molecular pattern molecules (DAMPs) [52], and tumor necrosis factor-related apoptosis-inducing ligand (TRAIL) [53]. The loss of* Gpx-1* exacerbated cigarette smoke-induced cell apoptosis in mice, suggesting that* Gpx-1* ^−/−^ genotype exacerbated cell death at least partially through the induction of the UPR. Our group has previously demonstrated that GPx-1 also regulates the activation of protein tyrosine phosphatase 1B (PTP1B) and protein phosphatase 2A (PP2A) [13]. Both of these phosphatases could impact smoke-induced cell survival [54, 55]. Equally, Nrf2 deficient cells undergo enhanced cell death following exposure to ER stress [47], which may be dependent on GPx-1 expression. Therefore, the data presented here suggests that enhanced CHOP expression in NHBE cells and mouse lungs may contribute to apoptosis. Other studies also suggest that certain elements of the UPR have several antiapoptotic and anti-inflammatory effects in other organs. XBP1 reduces CSE-induced CHOP and thereby is protected from CSE-induced apoptosis in a retinal pigment epithelia (RPE) cell line [56], via regulation of eIF2*α* and p38 phosphorylation [4]. Loss of CHOP expression exacerbated cell death through the downregulation of Nrf2 in RPE cells [4]. CHOP deficiency enhances apoptosis in hippocampal cells and impaired memory-related behavioural performances in mice with tunicamycin treatment [57]. Recently, deficiency of CHOP exaggerated lipopolysaccharide- (LPS-) induced inflammation and kidney injury in mice [58]. The importance of ER stress and the role of each member of the UPR in the development of lung disease still remain to be fully addressed. However, we have demonstrated that enhanced UPR coincides with worsening of symptoms that are countered with the expression of GPx-1 in mice.

## 5. Conclusion

Here we demonstrate that GPx-1 expression is reduced in NHBE cells isolated from COPD subjects, GPx-1 is a major regulator of the UPR under smoke exposure conditions, and acute ER stress induces lung GPx-1 expression. Together, our data indicate that the loss of GPX-1 expression in COPD lungs could contribute to disease progression by enhancing the UPR. These studies suggest that enhancing GPX-1 activity may be an effective therapeutic approach to prevent the damage induced by UPR in the lung.

## Figures and Tables

**Table 1:** Donor demographics for epithelial cell.

	Nonsmokers	Smokers	COPD donors
Number	9	6	9
Age (years)	36.3 ± 14.8	36.0 ± 13.2	49 ± 6.8
Gender (male/female)	2/7	3/3	5/4
Race (Caucasian/African American)	77.8%/22.2%	100%/0%	88.90%/11.1%
Pack years	0 ± 0	N/A	48.2 ± 14.5

Values are means ± SD. N/A = not available.

**Table 2:** TaqMan probe details for gene expression analysis.

Species	Gene target	NCBI reference sequence	TaqMan assay ID	Product size
Human	*DDIT3 (CHOP)*	NM_001195053.1	Hs00358796_g1	93
Human	*ATF4*	NM_001675.2	Hs00909569_g1	68
Human	*XBP1*	NM_001079539.1	Hs00231936_m1	60
Human	*HSPA5 (GRP78)*	NM_005347.4	Hs00607129_gH	146
Human	*HSP90B1 (GRP94)*	NM_003299.2	Hs00427665_g1	135
Human	*EDEM1*	NM_014674.2	Hs00976004_m1	89
Human	*GPX1 (GPx-1)*	NM_201397.1	Hs01028922_g1	70
Human	*IL-6*	NM_000600.3	Hs00985639_m1	66
Human	*ACTB*	NM_001101.3	Hs01060665_g1	63
Human	*PPFIA2 (CD45)*	NM_001220473.1	Hs00170308_m1	66
Human	*ITGAX (CD11C)*	NM_000887.3	Hs00174217_m1	119

Mouse	*Ddit3 (Chop)*	NM_001290183.1	Mm01135937_g1	92
Mouse	*Atf4*	NM_001287180.1	Mm00515325_g1	78
Mouse	*Xbp1*	NM_001271730.1	Mm00457357_m1	56
Mouse	*Hspa5 (Grp78)*	NM_001163434.1	Mm00517691_m1	75
Mouse	*Hsp90b1 (Grp94)*	NM_011631.1	Mm00441926_m1	67
Mouse	*Edem1*	NM_138677.2	Mm00551797_m1	63
Mouse	*Gpx-1*	NM_008160.6	Mm00656767_g1	134
Mouse	*Gpx-2*	NM_030677.2	Mm00850074_g1	147
Mouse	*Gpx-3*	NM_008161.3	Mm00492427_m1	99
Mouse	*Gpx-4*	NM_008162.3	Mm00515041_m1	103
Mouse	*Sod1*	NM_011434.1	Mm01344233_g1	71
Mouse	*Actb*	AK075973.1	Mm02619580_g1	143
